# Toward Biology-Driven Diagnosis of Atypical Parkinsonian Disorders

**DOI:** 10.3390/neurosci6040107

**Published:** 2025-10-21

**Authors:** Oscar Arias-Carrión, Elizabeth Romero-Gutiérrez, Emmanuel Ortega-Robles

**Affiliations:** 1 División de Neurociencias, Clínica, Instituto Nacional de Rehabilitación Luis Guillermo Ibarra Ibarra, Mexico City 14389, Mexico; romguteliz@gmail.com; 2 Tecnologico de Monterrey, Escuela de Medicina y Ciencias de la Salud, Mexico City 14380, Mexico

**Keywords:** progressive supranuclear palsy, corticobasal degeneration, multiple system atrophy, atypical Parkinsonism, biomarkers, diagnostic algorithms

## Abstract

Atypical parkinsonian disorders—progressive supranuclear palsy (PSP), corticobasal degeneration (CBD), and multiple system atrophy (MSA)—are rare, rapidly progressive neurodegenerative syndromes characterized by distinct molecular pathologies, heterogeneous clinical phenotypes, and limited therapeutic options. Accurate diagnosis remains a major clinical challenge, especially during early and prodromal phases, due to overlap with Parkinson’s disease (PD), phenotypic evolution, and the absence of reliable stand-alone biomarkers. Misclassification delays prognosis, impairs patient care, and hinders clinical trial design. This review synthesizes advances from 2015 to 2025 in clinical, imaging, and biomarker-based diagnosis of PSP, CBD, and MSA. We examine their phenotypic spectra, neuropathological substrates, and epidemiological trends, and critically evaluate the diagnostic performance and translational potential of emerging tools—including quantitative MRI morphometry, second-generation tau and α-synuclein PET ligands, neurophysiological markers such as video-oculography and autonomic testing, and fluid biomarkers such as neurofilament light chain. Persistent diagnostic barriers are identified, from phenotypic mimicry and pathological pleomorphism to the limited specificity of molecular assays and inequitable access to advanced technologies. We propose tiered, multimodal diagnostic algorithms that integrate structured clinical phenotyping with quantitative imaging, molecular diagnostics, systemic risk profiling, and autopsy-linked validation. Such biology-anchored approaches could enable diagnosis years before classical features emerge, improve patient stratification for disease-modifying trials, and lay the foundation for precision medicine in atypical parkinsonian disorders. A paradigm shift from descriptive nosology to mechanistically grounded frameworks is essential to accelerate early intervention and transform the clinical management of these devastating diseases.

## 1. Introduction

Parkinsonism is a clinical syndrome defined by bradykinesia with additional motor signs—rigidity, tremor, and postural instability—but its causes are heterogeneous. Parkinsonism is a clinical syndrome defined by bradykinesia accompanied by rigidity, tremor, or postural instability, yet its causes are highly heterogeneous. While idiopathic Parkinson’s disease (PD) accounts for most cases, a significant proportion arises from atypical parkinsonian disorders (APDs), notably progressive supranuclear palsy (PSP), corticobasal degeneration (CBD), and multiple system atrophy (MSA). These syndromes are biologically distinct—4-repeat (4R) tauopathies underpin PSP and CBD, whereas MSA is characterized by α-synuclein pathology—and they follow different trajectories, complications, and therapeutic priorities [1,2]. Early and accurate distinction from PD and from one another is therefore essential for prognosis, patient counselling, and the design of disease-modifying trials.

Over the past decade, diagnostic frameworks for APDs have advanced from purely clinicopathological descriptions to structured, phenotype-based criteria supported by neuroimaging, fluid biomarkers, and, increasingly, molecular insights. The 2017 Movement Disorder Society (MDS) criteria for PSP expanded diagnostic capture beyond Richardson’s syndrome by incorporating variant phenotypes characterized by ocular motor dysfunction, early postural instability, and frontal dysexecutive syndromes [1,3]. Similarly, the 2022 MDS criteria for MSA introduced a three-tiered classification—“clinically established,” “clinically probable,” and “possible prodromal”—integrating autonomic failure, Parkinsonism and/or cerebellar features, and characteristic MRI markers such as putaminal atrophy, middle cerebellar peduncle hyperintensity, and the “hot-cross-bun” sign [2]. CBD remains diagnostically challenging because corticobasal syndrome (CBS), its most frequent clinical presentation, is pathologically heterogeneous: Alzheimer’s disease (AD), PSP, or frontotemporal lobar degeneration (FTLD-TDP) frequently underlie CBS at autopsy [4,5]. This pleomorphism underscores the need for diagnostic approaches that integrate pathology-specific biomarkers to complement clinical phenotyping.

Supportive investigations have become increasingly central to diagnostic refinement. Quantitative MRI metrics such as the Magnetic Resonance Parkinsonism Index 2.0 (MRPI 2.0) significantly improve the differentiation of PSP, particularly the PSP–Parkinsonism subtype, from PD, and automated analysis pipelines are enhancing their reproducibility and accessibility [6,7,8]. Cardiac ^123^I-MIBG scintigraphy, typically reduced in PD but preserved in MSA, adds discriminatory value when interpreted alongside autonomic function testing [9]. Fluid biomarkers complement imaging findings: concentrations of neurofilament light chain (NfL) in cerebrospinal fluid and serum are consistently higher in APDs than in PD and correlate with disease progression, aiding triage and prognostication [10]. Moreover, seed-amplification assays for misfolded α-synuclein show high sensitivity in PD and dementia with Lewy bodies but are often negative in MSA—a biologically meaningful distinction that informs patient stratification in clinical trials [11,12].

Emerging evidence also points to systemic and vascular contributions to APD pathogenesis. Hypertension, diabetes, and small-vessel disease have been implicated in modulating disease onset and progression, particularly in CBS and PSP, highlighting the importance of considering comorbid pathologies during diagnosis [13,14,15]. In addition, the role of inflammatory and trophic markers—including glial cell line-derived neurotrophic factor (GDNF), interleukins, and hepcidin—is under investigation as potential biomarkers for differentiating PSP–Richardson’s syndrome from PSP–Parkinsonism [16,17,18].

Despite these advances, early-stage diagnosis remains challenging due to overlapping clinical features, evolving phenotypes, and the absence of stand-alone biomarkers. This review aims to address these gaps by synthesizing current evidence and proposing tiered diagnostic algorithms for PSP, CBD, and MSA. These structured approaches integrate sequential stages: (1) comprehensive clinical assessment, including ocular, cortical, cerebellar, and autonomic domains; (2) neurophysiological evaluation, such as video-oculography for saccadic slowing in PSP, cortical myoclonus studies in MSA, and somatosensory evoked potentials in CBS; (3) advanced structural and molecular imaging, including MRPI 2.0 and tau- or α-synuclein-sensitive PET; (4) fluid biomarker analysis, including NfL and multiplex panels; and (5) longitudinal reassessment to capture phenotypic evolution and refine diagnostic confidence.

Our goal is pragmatic and forward-looking: to shorten diagnostic latency, enable timely initiation of symptomatic and supportive interventions, and optimize patient selection for emerging disease-modifying therapies. Achieving this requires a paradigm shift from descriptive syndromic labelling to biology-driven, multimodal diagnosis, embedded within multidisciplinary clinical services. Such an approach aligns clinical, neuroimaging, molecular, and neurophysiological evidence to generate diagnostic certainty earlier in the disease course and to guide precision medicine strategies for these devastating neurodegenerative disorders.

## 2. Progressive Supranuclear Palsy (PSP)

Progressive supranuclear palsy is a primary 4R-tauopathy defined by the abnormal aggregation of tau protein in neurons and glia, producing hallmark lesions such as globose neurofibrillary tangles, coiled bodies, and tufted astrocytes. These pathologies predominantly involve the brainstem, basal ganglia, and associated white matter tracts, leading to characteristic motor, cognitive, and ocular motor dysfunctions [6,19,20]. Clinically, the classical phenotype—PSP–Richardson’s syndrome (PSP-RS)—is marked by early postural instability with backward falls, vertical supranuclear gaze palsy, axial-predominant rigidity, dysarthria, dysphagia, and a subcortical dysexecutive cognitive profile [1,3]. The global prevalence is estimated at around 7 per 100,000, with an incidence up to 2.6 per 100,000 person-years, onset typically in the sixth decade of life, and a median survival of 5–7 years [21,22,23]. Despite these distinctive features, diagnostic delays of 3–5 years are common, largely due to phenotypic overlap with PD and other atypical parkinsonian syndromes in early stages [24]. Addressing this gap requires structured diagnostic algorithms that integrate clinical, neurophysiological, imaging, molecular, and biomarker-based evidence.

### 2.1. Aetiology, Genetics, and Pathobiology

Most PSP cases are sporadic, but genetic susceptibility plays a substantial role. The *MAPT* H1 haplotype is the strongest known risk factor, while genome-wide association studies have identified additional loci, including *MOBP*, *EIF2AK3*, *STX6*, *SLCO1A2*, *DUSP10*, and *RUNX2*, as well as signals near *LRRK2*. Recent work has also implicated complement *C4A* and glial activation pathways, highlighting the central role of neuroinflammation in disease pathogenesis [25,26,27,28,29]. Rare familial forms linked to pathogenic *MAPT* mutations further expand the phenotypic spectrum but remain uncommon [30,31]. Beyond tau biology, mitochondrial dysfunction, oxidative stress, and innate immune activation contribute to disease mechanisms, suggesting that PSP arises from the convergence of proteostatic failure and glial–neuronal interactions [26,32].

Systemic factors such as hypertension, diabetes, and small-vessel disease may also influence PSP onset and progression [14]. Inflammatory and trophic mediators—including GDNF, interleukins, and hepcidin—are emerging as candidate biomarkers that could help discriminate between PSP-RS and PSP–Parkinsonism (PSP-P), underscoring the increasingly recognized interface between systemic inflammation and neurodegeneration [16,17,18].

### 2.2. Diagnostic Approach: From Clinical Suspicion to Biology-Driven Certainty

Early PSP is frequently misclassified as PD because of shared features such as bradykinesia and postural instability. However, PSP typically presents with symmetric, axial-predominant Parkinsonism, minimal or transient response to levodopa, paucity of rest tremor, and a characteristic evolution from vertical saccadic slowing to supranuclear gaze palsy with preserved vestibulo-ocular reflexes [1,33]. Pseudobulbar features—such as dysarthria and dysphagia—and early executive dysfunction further distinguish PSP from PD.

Neurophysiological testing provides additional diagnostic precision. Quantitative video-oculography can objectively document vertical saccadic slowing and increased square-wave jerks, features with high specificity for PSP. Combined amplitude–velocity metrics outperform single measures in differentiating PSP from PD [34,35,36]. These data underpin the 2017 MDS-PSP criteria, which stratify diagnostic certainty across classical and variant phenotypes [1,3].

**Figure 1:**
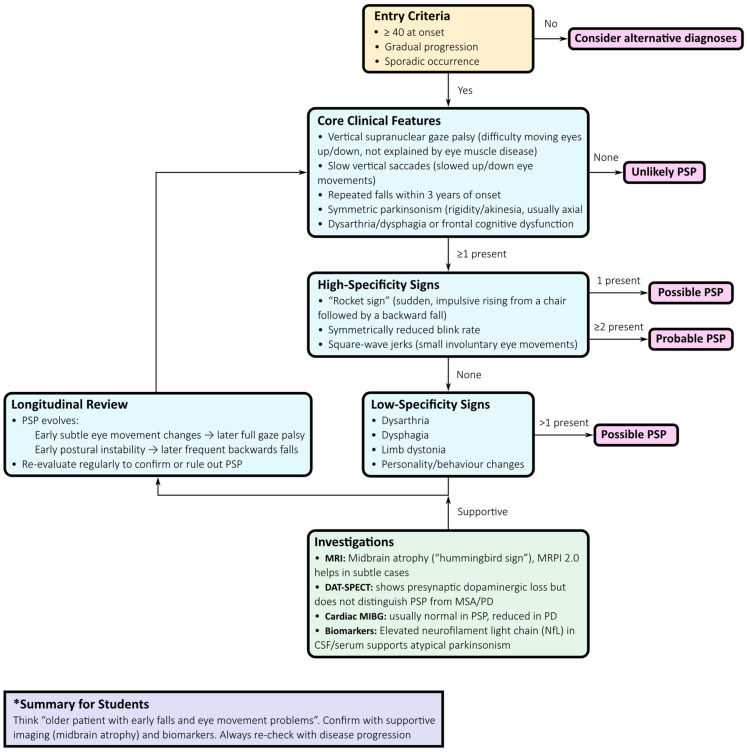
Stepwise Diagnostic Flowchart for Progressive Supranuclear Palsy (PSP). This flowchart outlines a structured diagnostic approach to progressive supranuclear palsy, integrating Movement Disorder Society (MDS) criteria with multimodal assessments to improve early detection and diagnostic confidence. Entry criteria are based on symmetric, axial-predominant Parkinsonism with vertical supranuclear gaze impairment, pseudobulbar features, and executive dysfunction. Ocular motor assessment, particularly vertical saccadic slowing and square-wave jerks, provides high specificity. Phenotypic classification captures classical PSP–Richardson’s syndrome and variant presentations such as PSP–Parkinsonism and PSP with predominant gait freezing. Supportive imaging markers include midbrain atrophy with pontine sparing (“hummingbird sign”) and quantitative indices such as MRPI 2.0. Ancillary investigations—including DAT-SPECT, cardiac ^123^I-MIBG scintigraphy, and fluid biomarkers such as neurofilament light chain—refine differential diagnosis. Longitudinal reassessment accounts for phenotypic evolution over time. Together, these steps enable a transition from descriptive syndromic diagnosis to biology-driven certainty, facilitating earlier intervention and clinical trial enrolment.

A structured, stepwise diagnostic algorithm is now recommended (Figure 1):

1. *Clinical evaluation:* Symmetric axial-predominant Parkinsonism, vertical gaze impairment, pseudobulbar features, and dysexecutive cognitive profile should prompt suspicion of PSP.
2. *Ocular motor assessment:* Slowing of vertical saccades progressing to supranuclear gaze palsy, with square-wave jerks, provides high specificity.
3. *Phenotypic classification:* The MDS criteria support early recognition of variant phenotypes, including PSP-P, PSP with predominant gait freezing (PSP-PGF), PSP–corticobasal syndrome (PSP-CBS), PSP–frontotemporal dementia (PSP-FTD), and language-predominant variants.
4. *Neuroimaging:* Midbrain atrophy with relative pontine sparing—the “hummingbird sign”—is a supportive feature. Quantitative measures such as the MRPI 2.0, increasingly available via automated pipelines, improve diagnostic accuracy, especially in distinguishing PSP-P from PD and MSA [7].
5. *Supportive imaging:* Dopamine transporter SPECT (DAT-SPECT) confirms presynaptic nigrostriatal degeneration but lacks nosological specificity. Cardiac ^123^I-MIBG scintigraphy, typically normal in PSP but reduced in Lewy body disorders, aids differential diagnosis, particularly with protocols incorporating salivary gland uptake [9].
6. *Fluid biomarkers:* NfL concentrations in cerebrospinal fluid or plasma are significantly higher in PSP than in PD and correlate with disease progression. Combined with MRI markers, NfL improves triage and prognostication [10].
7. *Longitudinal reassessment:* Regular re-evaluation is crucial, as phenotypes evolve and diagnostic certainty increases over time.

This multimodal approach reduces misclassification and exemplifies the shift from descriptive syndromic diagnosis to biology-anchored diagnostic certainty.

### 2.3. Phenotypic Spectrum and Evolution

Although PSP–Richardson’s syndrome is the archetypal presentation, several clinically relevant variants are now recognized. These include PSP–Parkinsonism (PSP-P), PSP with predominant gait freezing (PSP-PGF), PSP with corticobasal syndrome (PSP-CBS), PSP with frontotemporal dementia (PSP-FTD), and language-dominant variants such as progressive non-fluent aphasia. Phenotypic evolution over time is common, reinforcing the need for longitudinal follow-up rather than static diagnostic labels [1,33].

### 2.4. Investigations and Biomarkers

#### 2.4.1. MRI

Midbrain atrophy with relative pontine preservation (“hummingbird” profile) is supportive but not sufficient. Planimetric metrics markedly improve performance. The MRPI 2.0 distinguishes PSP—especially PSP-P—from PD and can be computed via validated automated pipelines, enhancing standardization across centers and supporting trials [6,7,8].

#### 2.4.2. Tau PET

Second-generation tracers show promise for 4R-tau imaging. Multicenter data with [^18^F]PI-2620 and [^18^F]florzolotau (APN-1607/PM-PBB3) demonstrate uptake patterns in the pallidum, subthalamus, and midbrain that complement MRI and may aid in early diagnosis and monitoring, although harmonized thresholds and head-to-head comparisons across 4R-tauopathies remain priorities [31,37,38,39].

#### 2.4.3. Dopaminergic Imaging and Autonomic Tracers

DAT-SPECT confirms presynaptic nigrostriatal denervation but does not reliably distinguish between PSP, PD, and MSA. Postsynaptic D2/3 imaging (e.g., ^123^I-IBZM) is no longer recommended for routine differential diagnosis due to limited added value [40]. Cardiac ^123^I-MIBG scintigraphy is typically normal in PSP and reduced in Lewy body disorders (PD/dementia with Lewy bodies); thus, it aids in the exclusion of PD when interpreted in the context of clinical findings, including newer protocols that incorporate salivary-gland uptake [41,42].

#### 2.4.4. Fluid Biomarkers

Neurofilament light chain in cerebrospinal fluid (CSF) or plasma is consistently higher in atypical Parkinsonism (including PSP) than in PD and correlates with progression; recent comparative studies show AUCs > 0.90 for PD vs. APD, supporting triage and prognostication rather than stand-alone diagnosis [43,44]. Combining NfL with quantitative MRI (e.g., midbrain measures, quantitative susceptibility mapping) improves classification in research settings [45].

#### 2.4.5. Neurophysiology

Blink reflex conditioning revealed marked abnormalities in brainstem circuitry, with PSP showing higher degrees of abnormality than CBS, suggesting its potential value for detecting brainstem dysfunction. Evoked potential studies demonstrated prolonged central conduction time and enlarged somatosensory-evoked potentials, as well as abnormal visual and brainstem auditory-evoked potentials, the latter correlating with fall risk in PSP. Event-related potential analyses found increased P300 latency and reduced amplitude, indicating cognitive slowing. Transcranial magnetic stimulation studies reported altered cortical inhibition, abnormal silent periods, and reduced short-latency afferent inhibition, reflecting impaired sensorimotor and cholinergic function. Moreover, theta-burst stimulation revealed abnormal motor cortex plasticity in PSP [46].

### 2.5. Neuropathology

Neuropathological confirmation remains the gold standard. PSP is defined by widespread 4R-tau deposition in neurons and glia, with selective vulnerability of the globus pallidus, subthalamic nucleus, substantia nigra, and brainstem tegmentum. Tufted astrocytes are pathognomonic and distinguish PSP from other 4R-tauopathies [19,32].

### 2.6. Treatment and Management

To date, no therapy has demonstrated disease-modifying efficacy in PSP. Clinical trials of coenzyme Q10 and the glycogen synthase kinase-3β inhibitor tideglusib did not meet primary endpoints despite early imaging signals [47,48]. Anti-tau monoclonal antibodies, including gosuranemab and tilavonemab, have failed to slow clinical progression in phase 2 trials [49], though newer agents such as bepranemab (UCB0107) continue to be evaluated for safety and target engagement.

Management remains multidisciplinary and symptom-focused. Levodopa may offer limited, transient benefit. Amantadine can help with gait freezing, while botulinum toxin addresses blepharospasm and eyelid-opening apraxia. Early speech and swallowing therapy, including proactive discussion of gastrostomy, is critical. Tailored physiotherapy and occupational therapy address falls and executive dysfunction, while targeted interventions for mood, sleep, and pseudobulbar affect improve quality of life [50]. Emerging evidence also suggests that vascular risk factor control and neuroinflammatory modulation could become adjunctive therapeutic targets [14].

### 2.7. Future Directions

The future of PSP diagnosis and management requires a paradigm shift from traditional, descriptive syndromic classifications to integrated, biology-driven frameworks that combine multimodal data. Advances in quantitative MRI and MRPI 2.0, second-generation tau PET tracers such as [^18^F]PI-2620 and [^18^F]florzolotau, neurophysiological testing (e.g., video-oculography and saccadic dynamics), and fluid biomarkers such as plasma or CSF NfL have already demonstrated complementary diagnostic value [1,7,22]. The integration of systemic inflammatory markers (e.g., GDNF, interleukins, hepcidin), vascular and metabolic risk profiling, and glial activation signatures could further refine early detection and stratification, paving the way for targeted disease-modifying interventions [16,17,18].

Moreover, large-scale genomic and transcriptomic studies continue to reveal novel susceptibility loci beyond *MAPT*, including *MOBP*, *EIF2AK3*, and complement pathways, suggesting that future diagnostic algorithms will need to incorporate polygenic and inflammatory risk profiling [26,29]. Such strategies, embedded within multidisciplinary clinical services and validated in autopsy-linked cohorts, will be critical for shortening diagnostic latency, improving prognostication, and facilitating patient stratification for clinical trials.

Finally, precision medicine approaches should extend beyond diagnosis to disease modification. Therapeutic strategies aimed at modulating tau aggregation, neuroinflammation, mitochondrial dysfunction, and systemic comorbidities represent the next frontier. Combining early diagnosis with targeted interventions—potentially years before classical motor signs emerge—offers the most promising path toward altering the course of PSP and related tauopathies.

## 3. Corticobasal Degeneration (CBD)

Corticobasal degeneration is a 4R-tauopathy characterized by widespread deposition of hyperphosphorylated tau in neurons and glia, forming astrocytic plaques, coiled bodies, and ballooned neurons. Pathological involvement is typically asymmetric, affecting frontoparietal cortex, basal ganglia, and related white matter tracts, consistent with the predominantly unilateral motor and cortical signs observed clinically [51,52]. Clinically, it is characterized by progressive, levodopa-resistant, asymmetric Parkinsonism with cortical signs, including limb apraxia, cortical sensory deficits, and alien limb phenomena [51,53]. However, the most common clinical manifestation, corticobasal syndrome, is pathologically pleomorphic and can result from CBD, Alzheimer’s disease, PSP, FTLD-TDP, or mixed pathologies [5]. Population-based prevalence estimates are scarce but consistently lower than for PSP or MSA, ranging from 0.8 to 25 per 100,000, with incidence rates between 0.03 and 0.8 per 100,000 person-years [22]; onset typically occurs in the early 60s, and median survival is approximately 6–8 years from symptom onset [52]. This clinicopathological dissociation underscores the need for probabilistic, multimodal diagnostic strategies and longitudinal reassessment rather than static diagnostic labels.

### 3.1. Etiology and Genetics

Most CBD cases are sporadic, but shared genetic susceptibility with other primary tauopathies is increasingly recognized. The *MAPT* H1 haplotype, a major risk factor for PSP, has also been associated with CBD [54]. Genome-wide association studies suggest overlapping genetic architecture between CBD, PSP, and certain forms of frontotemporal lobar degeneration (FTLD), implicating genes involved in microtubule stabilization, vesicle trafficking, and glial activation [55]. While familial CBD is rare, pathogenic *MAPT* mutations occasionally produce clinicopathological CBD, expanding the phenotypic range [56]. Mechanistically, CBD pathology converges on the abnormal aggregation of 4R-tau, dysregulation of proteostasis, and microglial activation; however, the molecular triggers remain unclear [57].

### 3.2. Diagnostic Approach

**Figure 2:**
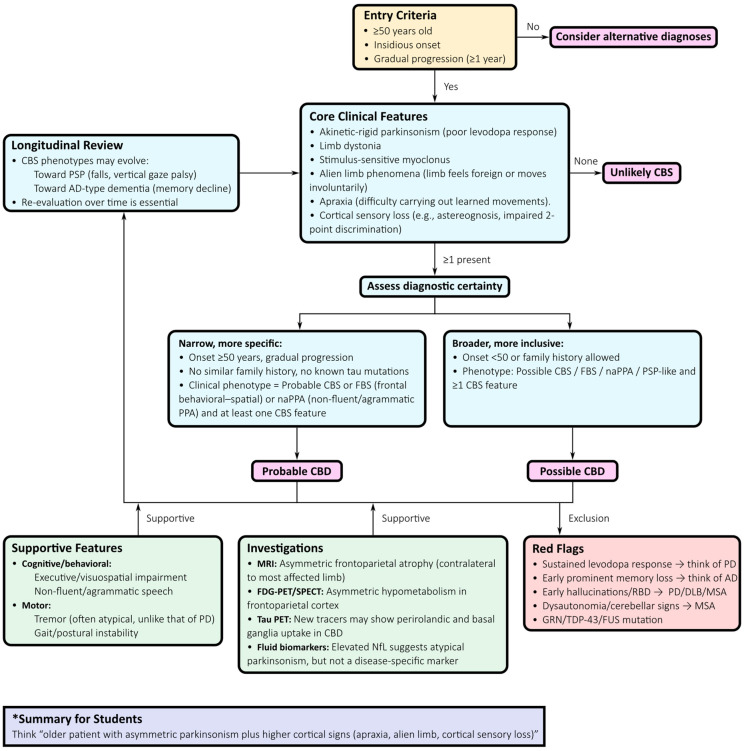
Diagnostic Algorithm for Corticobasal Syndrome (CBS) and Corticobasal Degeneration (CBD). This flowchart outlines a tiered diagnostic pathway for corticobasal syndrome and underlying corticobasal degeneration. The algorithm begins with the Armstrong clinical criteria, distinguishing “probable” from “possible” CBD based on asymmetric Parkinsonism, limb dystonia, myoclonus, and apraxia. Comprehensive neuropsychological assessment of executive, visuospatial, and language domains supports differential diagnosis, with non-fluent/agrammatic aphasia favouring CBD and early episodic memory loss suggesting Alzheimer’s disease–related CBS. MRI findings of asymmetric frontoparietal atrophy and functional imaging patterns on FDG-PET or perfusion SPECT strengthen clinico-anatomical correlations. Second-generation tau PET tracers demonstrate perirolandic and basal ganglia uptake in pathology-confirmed CBD. Fluid biomarkers, particularly elevated neurofilament light chain, improve prognostication when combined with imaging. Longitudinal follow-up captures phenotypic evolution toward PSP, behavioural-variant frontotemporal dementia, or posterior cortical atrophy. This probabilistic, multimodal approach reflects the shift from static labels to dynamic, biology-anchored diagnosis.

CBD most often manifests as corticobasal syndrome, a clinico-anatomical construct characterized by asymmetric akinetic-rigid Parkinsonism, limb dystonia, and stimulus-sensitive myoclonus. Here, we have expanded our discussion to outline a stepwise algorithm for CBD diagnosis (Figure 2). This approach integrates clinical features, neuropsychological testing, advanced neuroimaging, fluid biomarkers, and longitudinal follow-up to maximize diagnostic confidence:

1. *Application of Armstrong criteria:* Distinguishing between “probable” and “possible” CBD offers a standardized clinical framework. Although sensitivity and specificity are limited, they provide an initial scaffold for structured evaluation [51].
2. *Neuropsychological evaluation:* Comprehensive testing of executive, visuospatial, and language domains is essential. A non-fluent or agrammatic primary progressive aphasia profile favours CBD, whereas early episodic memory impairment points toward AD-related CBS [56].
3. *MRI assessment:* CBD classically presents with asymmetric frontoparietal atrophy contralateral to the most affected limb, sometimes involving the basal ganglia. While not diagnostic, these findings strengthen the clinicopathological correlation. Advanced morphometric methods, such as voxel-based morphometry or cortical thickness mapping, can detect early changes but lack individual-level specificity [6,58].
4. *Functional imaging:* FDG-PET and perfusion SPECT typically demonstrate asymmetric hypometabolism or hypoperfusion in the frontoparietal cortex and basal ganglia, often extending into the supplementary motor area, with relative sparing of the midbrain and cerebellum. These features distinguish CBD from PSP and MSA but overlap with AD-related CBS [59].
5. *Tau PET:* Second-generation tau tracers ([^18^F]PI-2620, [^18^F]florzolotau) demonstrate asymmetric perirolandic and basal ganglia uptake in pathologically confirmed CBD, complementing MRI findings and supporting early detection of 4R-tau pathology [60,61,62,63].
6. *Fluid biomarkers:* Elevated NfL is common and correlates with disease severity and progression. Combined with imaging and neuropsychological testing, it enhances discrimination between CBS due to CBD and CBS caused by AD or other pathologies [63,64,65].
7. *Longitudinal reassessment:* Phenotypic evolution is frequent, with progression toward PSP-like Richardson’s syndrome, behavioural variant FTD, non-fluent aphasia, or posterior cortical atrophy. Ongoing clinical review is essential, as early labels often evolve with disease progression.

This stepwise algorithm reflects a probabilistic diagnostic paradigm in which no single test is definitive. Instead, clinical suspicion anchored in structured criteria is refined through multimodal integration of imaging, biomarkers, and longitudinal follow-up.

### 3.3. Phenotypic Spectrum and Evolution

Pathologically confirmed CBD exhibits considerable phenotypic heterogeneity, manifesting as CBS, PSP-like Richardson’s syndrome, behavioural variant frontotemporal dementia, non-fluent/agrammatic primary progressive aphasia, or posterior cortical atrophy [52,53,66]. Conversely, CBS can result from diverse underlying pathologies, including AD, PSP, FTLD-TDP, or mixed forms [5,52,53,66]. Phenotypic evolution is common and clinically significant, reinforcing the need for longitudinal reassessment rather than single-timepoint diagnostic labelling.

### 3.4. Investigations and Biomarkers

#### 3.4.1. MRI

CBD typically shows asymmetric frontoparietal atrophy contralateral to the most affected limb, sometimes with basal ganglia atrophy [52,67]. Advanced morphometric analyses, such as voxel-based morphometry and cortical thickness mapping, may detect changes earlier but lack specificity at the individual level [6].

#### 3.4.2. FDG-PET and Perfusion SPECT

These modalities often reveal asymmetric hypometabolism or hypoperfusion in the frontoparietal cortex and basal ganglia, extending to the supplementary motor area, with relative sparing of the midbrain and cerebellum. This pattern supports differentiation from PSP and MSA but overlaps with AD-related CBS [53].

#### 3.4.3. Tau PET

First-generation tracers such as [^18^F]flortaucipir have shown variable binding in CBS/CBD and lower sensitivity for pure 4R-tau compared to AD-type mixed 3R/4R-tau. Second-generation tracers ([^18^F]PI-2620, [^18^F]florzolotau) display higher affinity for 4R-tau and, in pathologically confirmed CBD, show asymmetric uptake in perirolandic and basal ganglia regions that complement MRI findings [37,63].

#### 3.4.4. Fluid Biomarkers

No single CSF or plasma biomarker reliably distinguishes CBD from other tauopathies. NfL is often elevated, correlating with disease severity and rate of progression, and may aid in differentiating CBS due to CBD from CBS due to AD when combined with structural imaging and neuropsychological testing focused on language and executive function [43]. A recent study demonstrated that combining plasma NfL with p-tau181 enhances discrimination between CBD and AD-related CBS, refining biomarker-guided subtyping [63].

#### 3.4.5. Neurophysiology

Electromyography and reflex studies revealed abnormal C reflexes and long-latency reflexes even at rest, reflecting altered sensorimotor integration. EEG analyses demonstrated background slowing and reduced occipital alpha activity, while back-averaging techniques indicated a lack of jerk-locked cortical potentials, consistent with a subcortical origin of myoclonus. Somatosensory-evoked potentials are typically not enlarged in CBS, helping distinguish it from cortical myoclonus. Brainstem auditory-evoked potentials were frequently abnormal and correlated with brainstem atrophy, and event-related potentials showed increased P300 latency and reduced amplitude, paralleling cognitive dysfunction. Transcranial magnetic stimulation studies revealed abnormal motor cortex plasticity and altered intracortical inhibition, though findings have not yet reached clinical applicability [46].

### 3.5. Pathology

Neuropathology reveals widespread 4R-tau deposition in neurons and glia, with astrocytic plaques—particularly in the cortex and striatum—serving as a defining feature distinguishing CBD from PSP. Coiled bodies in oligodendrocytes and ballooned neurons further characterize the disease, and lesion distribution correlates with the asymmetric cortical and extrapyramidal signs seen clinically [51,52,53].

### 3.6. Treatment and Management

No disease-modifying therapies exist for CBD. Management is multidisciplinary and symptomatic. Levodopa trials can be considered, but often yield modest, short-lived benefits. Amantadine may alleviate gait freezing and other parkinsonian symptoms. Botulinum toxin can reduce focal dystonia, and myoclonus may respond to valproate or levetiracetam. Early speech-language therapy, occupational therapy for apraxia, and physiotherapy with fall-prevention strategies are critical to maintaining quality of life [4,67,68].

Given the frequent phenotypic overlap with other tauopathies and AD, multimodal diagnostic strategies—integrating detailed clinical characterization, structural and functional imaging, targeted biomarker analysis, and longitudinal follow-up—are essential for early and accurate recognition. Such approaches not only guide prognosis and patient counselling but also facilitate enrolment into clinical trials of tau-targeted therapies and other emerging interventions [6,37,43].

### 3.7. Future Perspectives

Future diagnostic strategies for CBD must move decisively from descriptive syndromic labelling to biology-led, multimodal frameworks. Given the pleomorphic nature of corticobasal syndrome, probabilistic diagnosis should integrate: (1) systemic risk profiling—hypertension, diabetes, and small-vessel disease, which can modify phenotype and rate of progression; (2) neuroinflammatory signatures—including astroglial and microglial markers; and (3) polygenic risk scores anchored to MAPT and emerging loci shared across 4R-tauopathies. These data should be combined with quantitative structural MRI, FDG-PET (perirolandic/frontoparietal hypometabolism), and second-generation tau PET (e.g., [^18^F]PI-2620, [^18^F]florzolotau), which show asymmetric perirolandic/basal ganglia uptake in pathology-confirmed CBD and complement MRI for early detection. Recent evidence from a data-driven classification study further supports this multimodal approach, showing that combining [^18^F]PI-2620 tau PET with plasma p-tau181 and NfL significantly improves differentiation between CBD and AD-related CBS [63]. Harmonized acquisition protocols, centre-independent thresholds, and autopsy-linked validation cohorts are now essential for clinical translation.

Equally important is acknowledging that CBS is a syndrome rather than a disease. Routine screening for Alzheimer’s co-pathology (amyloid and p-tau in CSF or plasma; amyloid/tau PET when available) should be embedded in diagnostic pathways to avoid misclassification and to enable stratified, pathology-specific clinical trials. Longitudinal re-evaluation must be standard, since phenotypes often evolve toward PSP-like Richardson’s syndrome, non-fluent/agrammatic primary progressive aphasia, behavioural-variant FTD, or posterior cortical atrophy. Together, these steps will accelerate precision medicine in CBD by aligning clinical phenotypes with underlying biology and trial-ready enrichment strategies.

## 4. Multiple System Atrophy (MSA)

Multiple system atrophy comprises two motor phenotypes—MSA-P (parkinsonian) and MSA-C (cerebellar)—but mixed features frequently emerge as disease progresses. Patients with MSA-P often later manifest limb/gait ataxia, while those with MSA-C commonly develop bradykinesia and rigidity. Subtype frequency varies geographically, with MSA-C predominating in East Asia and MSA-P more common in Western cohorts. A prodromal phase is increasingly recognized and codified in the MDS criteria: isolated autonomic failure (e.g., neurogenic orthostatic hypotension or urinary retention with large post-void residuals), polysomnography-confirmed REM sleep behaviour disorder, hyposmia in some individuals, and subtle motor abnormalities. Systematic identification of this phase is clinically relevant for surveillance, risk counselling, and trial enrolment in disease-modifying studies.

### 4.1. Etiology and Genetics

MSA is considered a primary oligodendrogliopathy, characterized by α-synuclein aggregation within glial cytoplasmic inclusions (Papp–Lantos bodies), which serves as the neuropathological hallmark [11]. The molecular basis remains incompletely understood. Rare pathogenic or risk variants in *COQ2*, a gene encoding a key enzyme in coenzyme Q10 biosynthesis, have been identified in Japanese and other Asian populations, particularly in MSA-C, and are associated with reduced tissue and plasma coenzyme Q10 levels [69,70]. Additional genetic studies have explored *SNCA* variants and other mitochondrial or lysosomal genes; however, no robust, population-wide associations have been established outside of COQ2 [71].

### 4.2. Diagnostic Approach

The diagnosis of MSA requires the integration of autonomic, motor, and supportive clinical features, as defined by the revised Movement Disorder Society (MDS) criteria [2]. Clinicians should suspect MSA when a characteristic combination of these features occurs, particularly in patients with rapid disease progression and limited response to dopaminergic therapy.

In the parkinsonian subtype (MSA-P), the syndrome is typically symmetric, with bradykinesia and rigidity but without the classic pill-rolling rest tremor of idiopathic Parkinson’s disease. Levodopa responsiveness, if present, is modest and transient. A jerky postural or action tremor—often associated with cortical myoclonus—is common [72]. In the cerebellar subtype (MSA-C), gait and limb ataxia predominate, accompanied by dysarthria and oculomotor abnormalities, with Parkinsonism typically emerging later in the disease course [73].

Autonomic failure is the hallmark diagnostic feature and may precede motor manifestations by several years. It encompasses neurogenic orthostatic hypotension, urinary urgency or retention with large post-void residual volumes, and erectile dysfunction in men [72]. Respiratory manifestations—particularly nocturnal inspiratory stridor caused by laryngeal abductor paresis—may occur early and are associated with a worse prognosis [74,75]. Supportive clinical features include REM sleep behaviour disorder, early postural instability, orofacial dystonia, and severe gait freezing, whereas early dementia or prominent visual hallucinations suggest alternative diagnoses such as dementia with Lewy bodies (DLB) [2,71].

**Figure 3:**
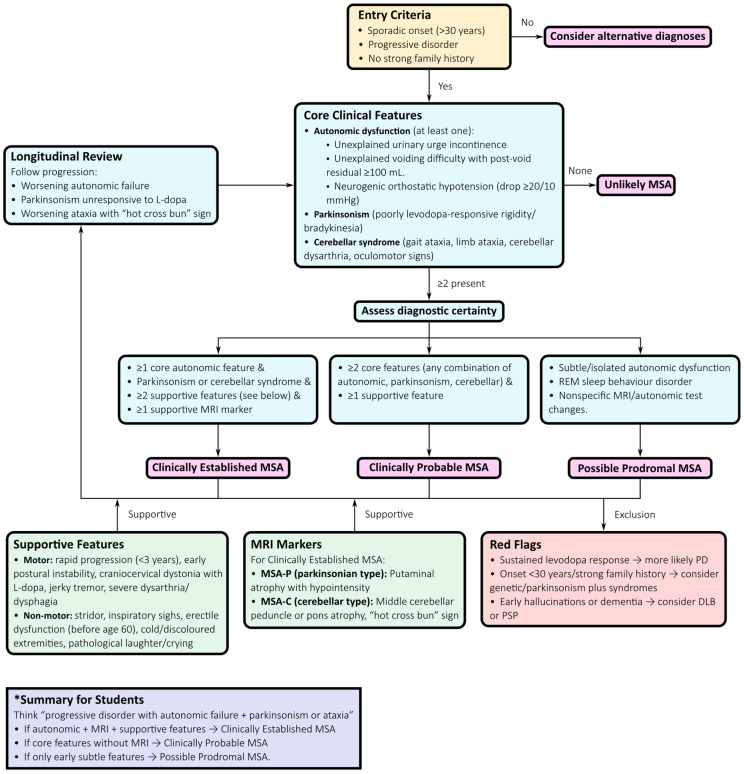
Diagnostic Flowchart for Multiple System Atrophy (MSA). This flowchart outlines a comprehensive diagnostic pathway for multiple system atrophy, structured according to the 2022 MDS criteria and emphasizing early recognition of prodromal and variant presentations. Entry features include autonomic failure—such as neurogenic orthostatic hypotension, urinary retention, and erectile dysfunction—which often precede motor manifestations by several years. Core domains include symmetric Parkinsonism with poor levodopa response (MSA-P) and cerebellar ataxia with dysarthria and oculomotor abnormalities (MSA-C). Supportive features include REM sleep behaviour disorder, early postural instability, and inspiratory stridor. Structural MRI markers—such as the “hot cross bun” sign in the pons, putaminal atrophy, and middle cerebellar peduncle hyperintensity—enhance diagnostic confidence, while quantitative morphometry improves early detection. Ancillary studies, including comprehensive autonomic testing, DAT-SPECT, FDG-PET, and cardiac ^123^I-MIBG scintigraphy, refine differential diagnosis. Serial assessments are essential, as isolated autonomic dysfunction may evolve into combined motor syndromes. This integrative algorithm exemplifies a biology-driven diagnostic framework that underpins precision medicine and trial readiness in MSA.

To enhance diagnostic accuracy and reduce delays, a structured, stepwise diagnostic algorithm is recommended (Figure 3):

1. *Initial presentation:* Suspect MSA when symmetric Parkinsonism (MSA-P) or cerebellar ataxia (MSA-C) occurs alongside autonomic failure. Autonomic symptoms frequently precede motor signs and should trigger early diagnostic consideration.
2. *Autonomic assessment:* Comprehensive autonomic testing—including tilt-table testing, Valsalva manoeuvre, and quantitative sudomotor axon reflex testing—documents cardiovascular and sudomotor involvement. Urodynamic studies can reveal detrusor overactivity with impaired contractility or detrusor–sphincter dyssynergia, providing strong diagnostic support [76,77,78].
3. *Motor examination:* Poor or transient levodopa response, jerky cortical myoclonus, or focal dystonia favour MSA over PD, where sustained levodopa responsiveness and a classic rest tremor are typical [72].
4. *MRI assessment:* Structural MRI findings such as the “hot cross bun” sign in the pons, putaminal atrophy and hypointensity, and middle cerebellar peduncle (MCP) hyperintensity or atrophy support the diagnosis. Quantitative morphometric measures, including MCP width and pons-to-MCP area ratios, further improve early diagnostic accuracy [6,79,80].
5. *Dopaminergic imaging:* DAT-SPECT confirms presynaptic dopaminergic degeneration but does not distinguish MSA from PD or PSP, serving primarily to establish the presence of neurodegeneration [80,81].
6. *Cardiac ^123^I-MIBG scintigraphy:* Cardiac sympathetic innervation is typically preserved or only mildly reduced in MSA but is markedly reduced in PD and DLB, helping differentiate synucleinopathies in the appropriate clinical context [80,82].
7. *FDG-PET:* Patterns of putaminal, pontine, and cerebellar hypometabolism with relative cortical sparing support MSA and assist in distinguishing it from PSP and PD [83].
8. *Pathology:* Definitive diagnosis is achieved by demonstrating α-synuclein–positive glial cytoplasmic inclusions (Papp–Lantos bodies) in oligodendrocytes, typically confirmed post-mortem [76,84,85].
9. *Longitudinal reassessment:* Because MSA phenotypes evolve over time, serial evaluations are essential. Patients initially presenting with isolated autonomic failure may later develop parkinsonian or cerebellar features, clarifying the diagnosis and informing prognosis and clinical trial eligibility [72,76,85].

This diagnostic algorithm underscores that early recognition of autonomic dysfunction, combined with supportive motor and imaging features, is central to identifying MSA. While classical MRI markers such as the “hot cross bun” sign remain highly supportive in the correct clinical context, diagnostic specificity is greatly enhanced when combined with quantitative imaging, FDG-PET, and fluid biomarkers such as NfL. Finally, longitudinal reassessment is critical to capture phenotypic evolution, particularly in cases transitioning from isolated autonomic dysfunction to combined motor syndromes.

### 4.3. Phenotypic Spectrum

Although MSA-P and MSA-C are the two recognized motor subtypes, patients often develop features of both forms as the disease progresses. MSA-P may later exhibit cerebellar signs, whereas MSA-C often evolves to include bradykinesia and rigidity [2,71]. The relative frequency of subtypes varies geographically, with MSA-C predominating in East Asia and MSA-P being more common in Western populations [2,71,85]. A prodromal phase, characterized by isolated autonomic failure, polysomnography-confirmed REM sleep behavior disorder, and subtle motor abnormalities, is increasingly recognized and is now a research category in the MDS criteria [2,71].

### 4.4. Investigations and Biomarkers

#### 4.4.1. MRI

Conventional imaging may reveal the “hot cross bun” sign in the pons, reflecting cruciform T2 hyperintensity due to selective pontocerebellar tract degeneration, as well as hyperintensity in the MCP with associated atrophy. In MSA-P, putaminal atrophy and posterolateral T2 hyperintensity with hypointense signal (“slit” sign) are common. Quantitative MRI morphometry, including MCP width and pons-to-MCP area ratios, improves early detection and reproducibility across centers [6].

#### 4.4.2. Functional Imaging

FDG-PET reveals hypometabolism in the putamen, pons, and cerebellum, with relative preservation of cortical metabolism—patterns that help distinguish MSA from PSP and Parkinson’s disease [86,87]. DAT-SPECT confirms presynaptic dopaminergic loss but cannot distinguish MSA from other degenerative Parkinsonisms [88]. Cardiac ^123^I-MIBG scintigraphy is usually normal or only mildly reduced in MSA, in contrast to the marked reduction seen in PD and dementia with Lewy bodies, aiding differential diagnosis in appropriate contexts [89].

#### 4.4.3. Autonomic Testing

Formal autonomic evaluation, including tilt-table testing, Valsalva maneuver, and quantitative sudomotor axon reflex testing, documents the severity and pattern of dysfunction. Urodynamic studies often reveal detrusor overactivity with impaired contractility or detrusor–sphincter dyssynergia [77,78].

#### 4.4.4. Fluid and Molecular Biomarkers

NfL concentrations in plasma and CSF are consistently higher in MSA than in PD and correlate with disease severity, rate of progression, and survival [90,91]. α-Synuclein seed amplification assays, while highly sensitive for Lewy body disorders, are less sensitive in MSA due to the distinct α-synuclein strains. However, ongoing refinements and alternative tissue sampling (e.g., skin) are improving sensitivity [11,92,93]. α-Synuclein PET tracers such as [^18^F]ACI-12589 have shown promising in vivo binding patterns in MSA and may enable pathology-specific diagnosis and staging in future clinical practice [94].

#### 4.4.5. Neurophysiology

Electromyography and reflex testing revealed distinctive patterns, such as anal sphincter denervation and reinnervation and abnormal bulbocavernosus reflexes, which are absent in Parkinson’s disease and reflect autonomic failure, a hallmark of MSA. Abnormal C reflexes and long-latency reflexes also indicate disrupted sensorimotor integration. EEG and evoked potential studies demonstrated that brainstem auditory-evoked potentials and pudendal SEPs are often altered and correlate with brainstem or autonomic dysfunction. Polysomnography consistently identifies REM sleep behavior disorder, a characteristic prodromal marker of α-synucleinopathies, being particularly prominent in MSA. Event-related potential studies found milder P300 abnormalities than in PSP or CBS, while Bereitschafts-potential recordings revealed reduced late components, particularly in the cerebellar subtype. Finally, transcranial magnetic stimulation investigations reported prolonged central motor conduction time and reduced corticospinal excitability, indicating motor pathway impairment [46].

### 4.5. Pathology

Neuropathology reveals widespread oligodendroglial cytoplasmic inclusions composed of misfolded α-synuclein, accompanied by neuronal loss and gliosis in the striatonigral and olivopontocerebellar systems [95]. Lesion distribution explains the distinct motor phenotypes and associated imaging signatures. Emerging work highlights oligodendroglial vulnerability, myelin dysfunction, and innate immune activation as convergent disease mechanisms [71,93].

### 4.6. Treatment and Management

There is no proven disease-modifying therapy for MSA. Parkinsonism may exhibit limited and transient benefit from levodopa, often constrained by the exacerbation of orthostatic hypotension. Management of neurogenic orthostatic hypotension involves fluid and salt supplementation, compression garments, head-up sleeping, and pharmacological agents such as midodrine, droxidopa, and, selectively, fludrocortisone [72]. Bladder dysfunction requires tailored interventions based on urodynamic findings, ranging from behavioral strategies to intermittent catheterization and intradetrusor botulinum toxin. Erectile dysfunction may respond to phosphodiesterase-5 inhibitors, provided that blood pressure is carefully monitored. Stridor is managed with early otolaryngology assessment, continuous positive airway pressure, or tracheostomy in severe cases, the latter associated with improved survival in selected patients [75]. REM sleep behavior disorder can be treated with melatonin or clonazepam. Multidisciplinary rehabilitation—including physiotherapy, occupational therapy, speech-language therapy, and nutritional support—should be initiated early to address gait instability, dysarthria, and dysphagia.

Several disease-modifying strategies are in clinical development. High-dose ubiquinol has shown modest slowing of motor decline in a phase 2 trial, supporting a possible role for coenzyme Q10 supplementation in genetically susceptible subgroups [69]. α-Synuclein immunotherapies, such as amlenotug (Lu AF82422), have demonstrated safety and target engagement in early-phase studies and are advancing to phase 3 evaluation [96]. The metal-binding modulator ATH434 has reported positive topline phase 2 results, with confirmatory trials underway [97]. As these therapies progress, the integration of the MDS diagnostic framework, quantitative imaging, and fluid biomarkers into recruitment strategies is becoming standard, allowing for earlier and biologically confirmed trial enrollment [2,71].

### 4.7. Future Perspectives

The future of MSA diagnosis and treatment lies in a decisive shift from traditional clinico-syndromic definitions toward integrated, biology-driven diagnostic and therapeutic frameworks. Despite advances in clinical criteria, diagnostic delays of 3–5 years remain common, and misclassification with Parkinson’s disease and PSP continues to impede clinical care and therapeutic development [2]. To overcome these barriers, future strategies must prioritize early, pre-symptomatic detection, pathology-specific biomarkers, and multimodal diagnostic platforms that combine neuroimaging, molecular assays, neurophysiology, and systemic risk profiling.

A crucial goal is the identification of prodromal MSA before motor or severe autonomic manifestations appear. Increasing evidence shows that isolated autonomic failure, polysomnography-confirmed REM sleep behaviour disorder, subtle motor dysfunction, or mild cognitive impairment can precede classical MSA by years [98,99]. Prospective longitudinal cohorts integrating these early features with fluid biomarkers—such as plasma and CSF NfL—could allow diagnosis at a stage when neuroprotective interventions might be most effective [90,91]. Combining NfL with plasma exosomal α-synuclein, markers of glial activation (e.g., soluble TREM2), and peripheral immune signatures may refine early detection and disease stratification.

The coming decade is likely to see transformative advances in molecular imaging. Early studies using α-synuclein–targeted PET ligands (e.g., [^18^F]ACI-12589) have demonstrated specific in vivo binding patterns distinct from those in Lewy body disorders, offering a pathway to pathology-based diagnosis and staging [94]. Parallel progress in quantitative MRI, including diffusion tensor imaging, susceptibility mapping, and automated morphometry of the middle cerebellar peduncles and basal ganglia, is improving diagnostic specificity and tracking of disease progression. In addition, functional connectivity mapping and network-based approaches may detect circuit-level changes before structural atrophy becomes apparent [6,80].

Advances in seed amplification assays hold promise for distinguishing α-synuclein strains associated with MSA from those in PD and DLB, addressing one of the current limitations of fluid biomarkers. Sensitivity is expected to improve further with novel amplification protocols, alternative biospecimens such as skin biopsies and olfactory mucosa, and multiplexed detection platforms that simultaneously profile α-synuclein, glial markers, and neurodegenerative signatures [11,92,93].

Growing evidence links systemic metabolic and vascular comorbidities, such as diabetes and hypertension, to accelerated disease progression and altered phenotypic expression in MSA [13]. Incorporating these risk profiles into diagnostic algorithms could improve prognostication and patient stratification. Moreover, genome-wide association studies have begun to identify potential susceptibility loci beyond SNCA, implicating genes related to lipid metabolism, glial function, and immune signalling [71,100]. As polygenic risk scores become more refined, they could complement clinical and biomarker data to enable precision risk prediction and guide personalized surveillance.

The absence of disease-modifying therapies underscores the need for biologically defined clinical trial cohorts. Future therapeutic strategies will increasingly rely on biomarker-based stratification to enrich trials for patients most likely to benefit. α-Synuclein immunotherapies such as amlenotug (Lu AF82422) and small-molecule aggregation modulators like ATH434 are advancing to phase 3 testing [97,101,102]. Other emerging approaches include oligodendroglial-targeted therapies, anti-inflammatory interventions, and gene-based therapies designed to modulate α-synuclein expression or glial stress responses [103].

A major shift in trial design is anticipated, moving from conventional clinical endpoints to biomarker-defined outcomes that capture disease biology and progression more sensitively. This approach will enable adaptive trial designs, where molecular responses—such as changes in α-synuclein PET signal, NfL trajectory, or glial activation markers—can serve as early efficacy readouts and guide dose optimization.

## 5. Discussion

Despite substantial advances in clinical criteria, imaging techniques, and biomarker discovery, the diagnosis of atypical parkinsonian disorders—notably progressive supranuclear palsy, corticobasal degeneration, and multiple system atrophy—remains challenging, particularly during the prodromal and early disease stages when disease-modifying interventions would be most effective [1,2,51]. Comparative analyses of their clinical, pathological, and imaging profiles underscore the persistent diagnostic uncertainty that arises from overlapping phenotypes, pathological heterogeneity, and incomplete biomarker validation (Table 1) [104]. Addressing these limitations requires a decisive paradigm shift from descriptive, syndromic classification toward biology-driven, multimodal diagnostic frameworks that integrate clinical phenotyping, neurophysiology, imaging, and molecular markers into harmonized algorithms.

A fundamental obstacle remains the phenotypic overlap between APDs and Parkinson’s disease in early disease stages. Bradykinesia, rigidity, and postural instability often predominate initially, leading to anchoring bias and delayed recognition of atypical features such as vertical supranuclear gaze palsy in PSP, asymmetric cortical sensory deficits in CBS, or early autonomic failure in MSA. As a result, diagnostic delays of three to five years are common. Embedding longitudinal re-evaluation protocols into care pathways—with predefined clinical milestones and triggers for ancillary testing—can reduce latency and improve diagnostic accuracy [24,105].

The introduction of the 2017 MDS-PSP and 2022 MDS-MSA criteria has broadened diagnostic capture by recognizing variant phenotypes and prodromal states [1,2]. However, this increased sensitivity comes at the cost of specificity in early disease. Variant presentations such as PSP–Parkinsonism or PSP–frontotemporal dementia are often misclassified, and the “possible prodromal” category for MSA awaits validation in pathology-confirmed cohorts. Future diagnostic frameworks must evolve into dynamic, “living criteria”—iteratively recalibrated using autopsy-verified data and incorporating quantitative imaging and biomarker thresholds to improve predictive accuracy over time.

CBS exemplifies the diagnostic challenge of pathological heterogeneity. Although frequently linked to CBD, CBS can also result from PSP, Alzheimer’s disease, FTLD-TDP, or mixed pathologies [5]. Without amyloid and phosphorylated tau assays or second-generation tau PET imaging, differentiating these causes remains unreliable. Incorporating amyloid-β and p-tau testing and reserving 4R-tau sensitive PET for suspected primary tauopathies will refine etiological classification, improve clinical trial stratification, and prevent misallocation of patients to inappropriate therapeutic interventions [106].

Conventional structural MRI remains an important diagnostic adjunct, but classical signs—such as midbrain atrophy (“hummingbird sign”) in PSP, the “hot cross bun” sign in MSA, or asymmetric frontoparietal atrophy in CBD—are supportive rather than diagnostic [6,80,107]. Quantitative measures such as the MRPI 2.0 significantly improve discrimination, particularly between PSP–Parkinsonism and PD [7]. Yet multicentre calibration, reproducible cut-offs, and open-source automated pipelines are essential to enable their widespread clinical use.

Molecular imaging is poised to transform the field. Second-generation tau PET tracers, including [^18^F]PI-2620 and [^18^F]florzolotau, reveal characteristic regional binding in PSP and CBD but require harmonized acquisition protocols and disease-specific thresholds for clinical implementation [31,37,38,39]. For MSA, α-synuclein PET imaging (e.g., [^18^F]ACI-12589) has shown the first pathology-specific signals in vivo [94]. However, larger, multicentre studies are needed to validate these findings and account for confounding factors such as co-pathology and off-target binding.

Dopaminergic imaging remains limited in its nosological resolution. While DAT-SPECT confirms presynaptic nigrostriatal degeneration, it cannot reliably differentiate PD from PSP or MSA and should be reserved for distinguishing degenerative from non-degenerative Parkinsonism [40]. Conversely, autonomic and peripheral sympathetic markers are underutilized but offer valuable diagnostic clues. Cardiac ^123^I-MIBG scintigraphy, typically normal in MSA but reduced in Lewy body disorders, aids differentiation in the appropriate clinical context [9,82,89]. Comprehensive autonomic testing—including tilt-table testing, Valsalva manoeuvre, and quantitative sudomotor axon reflex testing—should be incorporated into early diagnostic workups where available [76,77,78].

Fluid biomarkers offer critical complementary information. Plasma and CSF NfL levels are consistently elevated in APDs compared with PD and correlate with disease progression, but they lack pathological specificity [10,90,91]. Seed amplification assays can detect misfolded α-synuclein and discriminate Lewy body disorders but are often negative in MSA due to distinct α-synuclein strains [11,92,93]. Multiplex biomarker panels integrating NfL with markers of astrocytic activation (e.g., GFAP) and microglial response (e.g., sTREM2) may enhance differential diagnostic accuracy [11,64,108,109].

Neurophysiological investigations have contributed significantly to understanding disease mechanisms across APDs, revealing characteristic abnormalities in sensorimotor integration, cortical excitability, and brainstem function. Techniques such as transcranial magnetic stimulation, event-related and evoked potentials, and reflex conditioning paradigms have uncovered distinctive alterations in cortical inhibition, conduction time, and plasticity patterns that mirror disease-specific pathophysiology. However, according to the position paper by Suppa et al. [46], no neurophysiological test currently fulfils the criteria for standardized clinical application in the diagnosis of PSP, CBS, or MSA. Although some approaches—such as blink reflex conditioning and polysomnography for REM sleep behavior disorder—show potential clinical value in detecting brainstem dysfunction or prodromal α-synucleinopathy, they remain supportive rather than diagnostic tools. Other modalities, including cortical excitability measures or event-related potentials, have demonstrated group-level abnormalities but lack the sensitivity, specificity, and reproducibility required for diagnostic implementation. Standardization of protocols, normative datasets, and multicenter validation are necessary before these techniques can transition from research to clinical use.

The scarcity of autopsy-confirmed datasets and reliance on clinical diagnoses introduce verification bias, limiting the external validity of diagnostic algorithms. Establishing longitudinal, pathology-linked cohorts with interim biospecimen collection is critical to defining reliable diagnostic performance metrics [2]. Moreover, disparities in access to advanced diagnostics—such as tau PET, quantitative MRI, or autonomic laboratories—exacerbate global inequities in early diagnosis and trial participation. Hub-and-spoke referral networks, shared imaging interpretation platforms, and minimal diagnostic panels tailored for resource-limited settings could mitigate these barriers. Machine learning applied to harmonized multimodal datasets offers additional potential to enhance early classification, provided that rigorous external validation and bias mitigation strategies are implemented [110,111].

Future diagnostic pathways should embed tiered, multimodal algorithms that build upon MDS criteria but incorporate imaging, oculomotor and autonomic testing, and molecular biomarkers. For PSP and CBD, integrating MRPI 2.0 with 4R-tau sensitive PET and NfL could enable diagnosis years before classical signs emerge. For MSA, combining preserved cardiac ^123^I-MIBG uptake, characteristic MRI findings, elevated NfL, and emerging α-synuclein PET holds similar promise. CBS should routinely undergo screening for AD co-pathology using amyloid and p-tau markers, with tau PET reserved for suspected primary tauopathies.

Ultimately, a paradigm shift from descriptive syndromic labelling to biologically anchored diagnosis is essential. Achieving this goal will require large-scale, autopsy-verified, longitudinal cohorts; standardization of imaging and biomarker protocols; and equitable implementation across diverse healthcare settings. This biology-driven approach offers the most promising route to reducing diagnostic latency, enriching clinical trials with precisely characterized participants, and delivering earlier, personalized interventions that align with the mechanistic underpinnings of each disorder.

## Figures and Tables

**Table 1:** Differential Diagnosis: Parkinson’s Disease vs. Atypical Parkinsonisms.

Feature	Progressive Supranuclear Palsy (PSP)	Corticobasal Syndrome (CBS/CBD)	Multiple System Atrophy (MSA)	Parkinson’s Disease (PD)
Prevalence	~7 per 100,000	~4 per 100,000	~4 per 100,000	Much higher than atypical Parkinsonisms
Onset Age	~63 years	>60 years, variable	53–55 years	Typically > 60 years
Motor Symptoms	Symmetric Parkinsonism, early axial rigidity, backwards falls	Asymmetric rigidity, dystonia, myoclonus, apraxia	MSA-P: symmetric Parkinsonism (poor levodopa response); MSA-C: cerebellar signs (ataxia, dysarthria)	Asymmetric Parkinsonism, classic rest tremor, shuffling gait
Ocular Signs	Vertical supranuclear gaze palsy, slow vertical saccades	Difficulty initiating voluntary saccades, gaze apraxia	Rare, nonspecific ocular signs	Rare ocular involvement
Cognitive Profile	Subcortical dementia (executive dysfunction, slowed processing)	Frontal-executive and parietal dysfunction; alien limb; cortical sensory loss	Cognitive impairment may occur, but not early or prominent	Cognitive impairment usually occurs late (dementia in advanced PD)
Autonomic Dysfunction	Not prominent early; may appear late	Rare or mild	Prominent: orthostatic hypotension, urinary incontinence/retention, erectile dysfunction, constipation	Mild compared with MSA
Key Pathology	4R-tauopathy (globose tangles, tufted astrocytes)	4R-tauopathy (astrocytic plaques, ballooned neurons)	α-synucleinopathy (glial cytoplasmic inclusions)	α-synucleinopathy (Lewy bodies)
Imaging	Midbrain atrophy (“hummingbird sign”); increased MRPI 2.0	Asymmetric frontoparietal atrophy (contralateral to the affected limb)	“Hot cross bun” sign (pons), putaminal atrophy/hypointensity	Often, normal or nonspecific changes
Levodopa Response	Poor, transient at best	Poor or absent	Poor or transient (rare sustained response)	Good, especially early
Other Key Features	Early falls (<3 yrs), pseudobulbar palsy, reduced blink, square-wave jerks	Alien limb, cortical sensory loss, asymmetric apraxia	Early stridor, rapid progression, cold extremities, REM sleep behavior disorder	Classic pill-rolling tremor, clear honeymoon response to levodopa
Teaching tip for students	Falls and eye movement problems early	Asymmetric Parkinsonism with cortical signs (apraxia, alien limb phenomenon).	Autonomic failure plus Parkinsonism/ataxia	Asymmetric, tremor-dominant, levodopa-responsive

## Abbreviations

The following abbreviations are used in this manuscript:

α-syn	Alpha-synuclein
^123^I-IBZM	[^123^I]Iodobenzamide
^123^I-MIBG	[^123^I]Metaiodobenzylguanidine
4R-tau	4-repeat tauopathy
AD	Alzheimer’s disease
APD	Atypical parkinsonian disorder
AUC	Area under the receiver operating characteristic curve
CBS	Corticobasal syndrome
CBD	Corticobasal degeneration
CSF	Cerebrospinal fluid
DAT-SPECT	Dopamine transporter single-photon emission computed tomography
FDG-PET	[^18^F]Fluorodeoxyglucose positron emission tomography
FTD	Frontotemporal dementia
FTLD	Frontotemporal lobar degeneration
FTLD-TDP	FTLD with TPD-43-immunoreactive pathology
GSK-3β	Glycogen synthase kinase-3 beta
MCP	Middle cerebellar peduncle
MDS	Movement Disorder Society
MRI	Magnetic resonance imaging
MRPI 2.0	Magnetic Resonance Parkinsonism Index 2.0
MSA	Multiple system atrophy
MSA-C	Multiple system atrophy, cerebellar phenotype
MSA-P	Multiple system atrophy, parkinsonian phenotype
NfL	Neurofilament light chain
PD	Parkinson’s disease
PET	Positron emission tomography
PSP	Progressive supranuclear palsy
PSP-CBS	PSP with corticobasal syndrome
PSP-FTD	PSP with frontotemporal dementia
PSP-P	PSP–Parkinsonism
PSP-PGF	PSP with predominant gait freezing
PSP-RS	PSP–Richardson’s syndrome
